# Mouse CD163 deficiency strongly enhances experimental collagen-induced arthritis

**DOI:** 10.1038/s41598-020-69018-7

**Published:** 2020-07-24

**Authors:** Pia Svendsen, Anders Etzerodt, Bent W. Deleuran, Søren K. Moestrup

**Affiliations:** 1 Department of Molecular Medicine, University of Southern Denmark, Odense, Denmark; 2 Department of Clinical Medicine, Aarhus University Hospital, Aarhus, Denmark; 3 Department of Biomedicine, Aarhus University, Aarhus, Denmark; 4 Department of Rheumatology, Aarhus University Hospital, Aarhus, Denmark; 5 Department of Clinical Biochemistry, Aarhus University Hospital, Aarhus, Denmark

**Keywords:** Protein transport, Autoimmunity

## Abstract

The scavenger receptor CD163 is highly expressed in macrophages in sites of chronic inflammation where it has a not yet defined role. Here we have investigated development of collagen-induced arthritis (CIA) and collagen antibody-induced arthritis (CAIA) in CD163-deficient C57BL/6 mice. Compared to wild-type mice, the CIA in CD163-deficient mice had a several-fold higher arthritis score with early onset, prolonged disease and strongly enhanced progression. Further, the serum anti-collagen antibody isotypes as well as the cytokine profiles and T cell markers in the inflamed joints revealed that CD163-deficient mice after 52 days had a predominant Th2 response in opposition to a predominant Th1 response in CD163+/+ mice. Less difference in disease severity between the CD163+/+ and CD163−/− mice was seen in the CAIA model that to a large extent induces arthritis independently of T-cell response and endogenous Th1/Th2 balance. In conclusion, the present set of data points on a novel strong anti-inflammatory role of CD163.

## Introduction

The scavenger receptor CD163 is expressed exclusively in cells of monocytic origin with a high expression in M2-type macrophages where it has an established role in scavenging hemoglobin (Hb) released into plasma^1^. The receptor and its function have been most intensively studied in human systems, but the selective myelomonocytic expression of CD163 with a high upregulation in the M2-type macrophages is also seen in animals including rodents^2,3^. Expression of CD163 in human monocytes is low and in circulating mouse monocytes it is absent^4^. In humans, the complex formation of Hb and haptoglobin (Hp) leads to a high-affinity interaction with CD163^1^ and subsequent endocytosis and degradation of Hb-Hp into amino acids and the heme-derived anti-inflammatory metabolites CO and bilirubin^5^. Further, CD163 is reported to mediate uptake of tumor necrosis factor-α (TNF-α)-like weak inducer of the apoptosis (TWEAK)^6^ and High-Mobility Group Box 1 Protein (HMGB1) complexed to Hp^7^. The ligand repertoire disclosed so far points to an overall anti-inflammatory role of CD163, which is also supported by the features regulating CD163 expression. Mediators with a predominant anti-inflammatory activity such as glucocorticoid, interleukin (IL)-6 and IL-10 upregulate CD163, whereas compounds with a predominant pro-inflammatory activity such as lipopolysaccharide (LPS), TNF-α, interferon γ (INF-γ), CXC-chemokine ligand 4 (CXCL4), and granulocyte–macrophage colony-stimulating factor (CSF-2) downregulate CD163 expression^5^. During the acute pro-inflammatory response, CD163 in macrophages is also instantly down-regulated by ADAM17-induced shedding leading to a strong increase of soluble CD163 in plasma together with pro-inflammatory cytokines such as TNF-α that is also released by the metalloproteinase ADAM17^8–10^.

In many chronic inflammatory diseases, such as rheumatoid arthritis (RA), CD163 is highly up-regulated^5,11^ at the sites inflammation, which is in line with clinical evidence^12^ showing that accumulation of M2-type macrophages is an important part of the chronic and late inflammatory response. In mice, CD163 is mainly expressed by tissue-resident macrophages^4^ and recently CD163 was also shown to be expressed on a RELM-α positive subset of interstitial macrophages in the joint synovial membrane^13^. However, expression of CD163 on infiltrating inflammatory macrophages in mouse models of rheumatoid arthritis has not yet been described.

The M2 macrophage phenotype represents a broad spectrum of macrophage subtypes that are regarded as having mainly an anti-inflammatory role, which includes stimulation of tissue repair and to some extent counteracting the pro-inflammatory immune cells such as M1-type macrophages, B- and T cells and granulocytes^14,15^. It should therefore be noted that the macrophage M1/M2 paradigm is a simplified model of macrophage polarization rather than an exact description of the many overlapping classes of macrophages and their role in inflammation in vivo and the use of the nomenclature is therefore much debated^16^.

In this study, we analyzed the effect of CD163 deficiency in experimental mouse CIA and CAIA, which are well-known mouse models showing pathogenic features similar to RA such as pannus formation, cellular infiltration, synovitis and cartilage/bone destruction^17–20^. The CIA model, which is dependent on T and B cells, was used to examine T cell responses in CD163-deficient mice. Various mice strains display different susceptibility to development of arthritis and C57/BL6 mice, which represent the predominant mouse strain used for targeted gene disruption, has a lower arthritis response to collagen immunization compared to for instance DBA/1^21^. This difference in disease severity between mouse strains of different genetic background has been linked to different MHC class subtypes and the balance between Th1 and Th2 responses^20,22,23^. The Th1/Th2 balance is affected by the M1/M2 macrophage balance and vice versa^24^. In vitro, Th1 and Th2 cytokines stimulate polarization into the M1 and M2 macrophage phenotypes. In the present study, we have observed and described profound effects on the Th1/Th2 balance and arthritis severity in collagen-immunized C57/BL6 mice, when M2 macrophages become deficient of one of their major plasma membrane components, the receptor CD163.

## Results

### CD163 deficiency strongly enhances disease severity in experimental CIA

**Figure 1:**
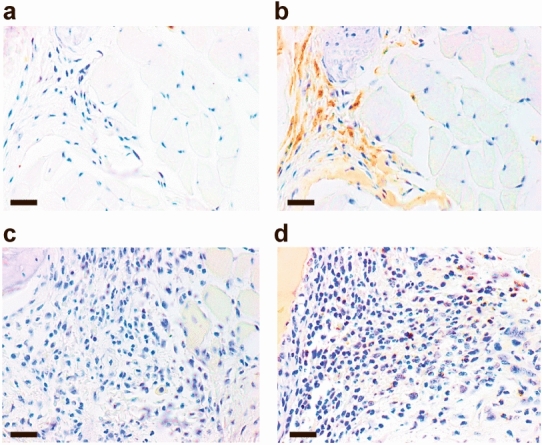
Representative anti-CD163 staining of inflamed paws of wildtype (**a**,**b**) and CD163-deficient CIA-mice (**c**,**d**). Rabbit IgG isotype controls (**a**,**c**) showed no staining, whereas the anti-CD163 antibody (**b**,**d**) specifically stained macrophages in wild type mice, but not in CD163-deficient mice. N = 5 for each group. Magnification × 20. Scale bars equals 50 µm.

**Figure 2:**
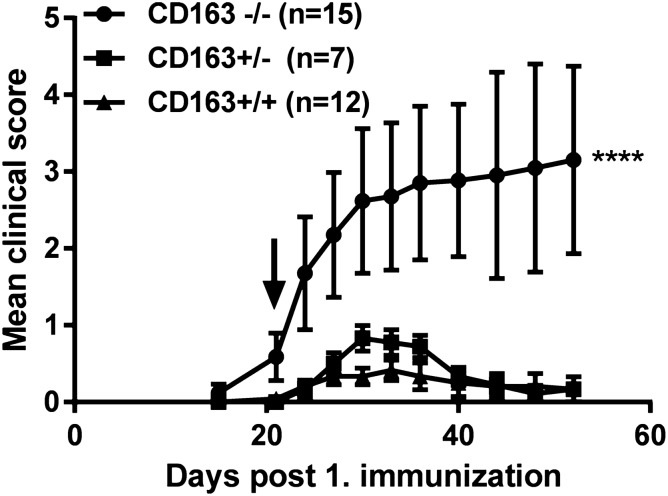
Induction of CIA in wild-type (n = 12), heterozygous (n = 7) and CD163−/− mice (n = 15). Clinical scores following immunization with chicken type II collagen in Freund’s complete adjuvant. Arrow indicates 2. immunization. Data presented as mean ± SEM. Paired t-test for significance; ****p < 0.0001. The figure is representative of three independent experiments.

Mice immunized with chicken type II collagen in adjuvant induced a modest arthritis response in normal C57/BL6 mice as previously reported^17,18^ (Figs. 1 and 2). In wild-type mice, CD163 macrophages were detected locally in the inflamed paws (Fig. 1b). In contrast, litter-mates deficient of CD163 expression (CD163 -/-) (Fig. 1d) displayed a much more severe arthritis with early onset and much prolonged response (Fig. 2; *p* < 0.0001). In terms of maximal clinical score, the severity of arthritis of the CD163 deficient mice was more than three-fold higher. After two months, the CD163−/− mice showed no signs of disease regression, whereas the CD163+/+ mice that peaked with disease activity around day 30 had no or only weak signs of disease (Fig. 2). Heterozygous mice (CD163+/−) were not significantly affected (Fig. 2; *p* = 0.08). Disease incidence (70–80%) was similar in in wild-type, CD163+/− and CD163−/− mice.

### Altered antibody isotype response against type II collagen in CD163-deficient mice

**Figure 3:**
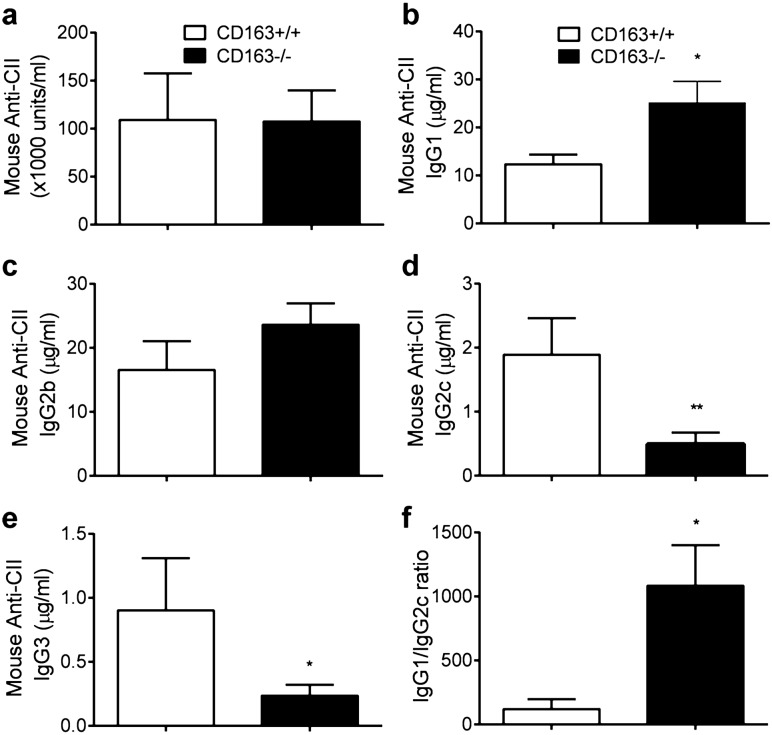
Anti-collagen type II antibody serum levels in the CIA model of CD163+/+ (n = 11) and CD163−/− mice (n = 13). (**a**) Total IgG (**b**) IgG1, (**c**) IgG2b, (**d**) IgG2c, (**e**) IgG3 and (**f**) IgG1/IgG2c ratio in serum were measured by an enzyme-linked immunosorbent assay at termination of the experiment (52 days post 1. immunization). Values are means ± SEM. Unpaired t-test performed for significance;*p < 0.05, **p < 0.01 versus wildtype.

High levels of circulating autoantibodies to CII are always associated with CIA and the IgG isotype profile against the endogenous mouse collagen antigen highly depends on the Th1/Th2 balance^25^. IgG2 and IgG3 antibodies are associated with a Th1 response, whereas IgG1 production is associated with a Th2 response^26,27^. Determination of the anti-collagen-II IgG1, IgG2b, IgG2c and IgG3 isotypes in the CD163−/− and CD163+/+ mice at termination of the CIA experiment (Fig. 3) revealed a similar quantitative collagen-specific IgG response in wild-type and CD163−/− mice (Fig. 3a), but with clear differences in the IgG subtypes. The titer of IgG1 was substantially and significantly higher in the CD163−/− mice (Fig. 3b; *p* = 0.032; CD163+/+ 12.32 ± 2.04 µg/ml, CD163−/− 25.11 ± 4.49 µg/ml), whereas the titers of IgG2c and IgG3 were significantly lower in the CD163−/− mice than in wild-type mice (Fig. 3d; *p* = 0.006; CD163+/+ 1.89 ± 0.57 µg/ml, CD163−/− 0.49 ± 0.18 µg/ml and Fig. 3e; *p* = 0.019; CD163+/+ 0.90 ± 0.41 µg/ml, CD163−/− 0.23 ± 0.08 µg/ml). Accordingly, the IgG1/IgG2c ratio was much higher in the CD163−/− mice compared to wild-type mice (Fig. 3f; *p* = 0.02; CD163+/+ 118.8 ± 77.9, CD163−/− 1,083 ± 317.5), indicating a prevalent systemic Th2 response and dampened Th1 response in the CD163−/− mice compared to the wild-type mice.

### Cytokine and chemokine profiles in the inflamed paws of collagen-immunized mice

Gene expression profiling of inflamed paws showed significant gene expression differences between the CD163−/− and CD163+/+ mice (Table 1). Several cytokine and chemokines genes involved in the inflammatory signaling cascade during development of human arthritis were up- or down-regulated in CD163−/− mice compared to the wildtype mice (Table 1). A prominent feature was down-regulation of gene expression of a number of RA- and CIA-associated Th1 cytokines including IL-17A, IL-23 and CSF-2 indicating that the increased arthritis disease was not driven by development of autoreactive Th17 cells and an enhanced Th17 response^28^. This interesting observation was further validated by immunohistochemical analysis of inflamed paws of CIA mice showing a significant lower accumulation of IL17-producing CD4 cells in the inflamed area in the CD163−/− mice compared to CD163+/+ mice (Supplementary Fig.S1; *p* = 0.011). RNA expression of TNF-α, a key pro-inflammatory cytokine in RA and CIA, was up-regulated locally in the paws of the CD163−/− mice (Table 1). Of note, the RNA expression of Th2 related chemokine (C–C motif) ligand 1 (CCL1), CCL20 and CCL22 (MIP3a) was also increased. The complete panel of the 92 investigated genes is shown in Supplementary Table S1.

**Table 1:** Genes significantly up- or down-regulated > twofold in paws of CD163−/− compared to CD163+/+ mice with or without CIA.

Pathway/ immune responses^a^	Gene description	Gene symbol	CIA CD163+/+ (n = 12) vs CIA CD163−/− (n = 15)	Naïve CD163+/+ (n = 8) vs Naïve CD163−/− (n = 8)
			Fold regulation	Fold regulation
	Aminoacyl tRNA synthetase complex	Aimp2	− 4.9*	
	Bone morphogenetic protein 2	BMP2	− 5.5*	
M2,Th2 related	Chemokine (C–C motif) ligand 1	Ccl1	+ 2.5*	
M1,Th2 related	Chemokine (C–C motif) ligand 11	Ccl11	− 4.5*	
M2,Th2 related	Chemokine (C–C motif) ligand 20	Ccl20	+ 12.1*	
M2,Th2 related	Chemokine (C–C motif) ligand 22	Ccl22	+ 2.2*	
M2,Th2 related	Chemokine (C–C motif) ligand 24	Ccl24	− 7.5*	
	Chemokine (C–C motif) ligand 6	Ccl6	− 4.0*	
Th2 related	Chemokine (C–C motif) receptor 8	Ccr8	+ 3.9*	
M2	Colony stimulating factor (M-CSF))	Csf1	− 3.9*	
M1,Th17 related	Colony stimulating factor 2 (GM-CSF)	Csf2	− 48*	
M1, Th1 related	Chemokine (C-X3-C motif) ligand 1	Cx3cl1	− 4.6*	
M2	Chemokine (C-X-C motif) ligand 12	Cxcl12	− 3.5*	
Th1 marker	Chemokine (C-X-C motif) receptor 3	Cxcr3	− 7.5*	
Th1 marker	Interferon gamma	Ifng	− 60*	− 7.7
Th2 related	Interleukin 10 receptor, beta	Il10rb	− 19.5*	
Th1 related	Interleukin 12 subunit alpha	IL12a	− 6.5	
CD4 + T cell marker	Interleukin 15	Il15	− 7.1*	
Th17,Th1 related	Interleukin 17 subunit alpha	Il17a	− 113*	
Th17, Th1 related	Interleukin 17 subunit beta	Il17b	5.4*	
Th17, Th1 related	Interleukin 23 subunit alpha	IL23a	− 61*	− 7.7
Th1 responses	Interleukin 27	Il27	− 6.8**	
Th1,Th2dif	Interleukin 2 receptor, alpha	IL2ra	+ 4.2*	
Th2 related	Interleukin 3	Il3	− 83.3*	
Th2 related	Interleukin 33	Il33	2.7*	
Th2 related	Interleukin 5	Il5	− 244*	− 7.5
Th2 related	Interleukin 5 receptor, alpha	Il5ra	− 6.1*	
Th1,Th2, Th17	Interleukin 7	Il7	+ 3.2*	
Th1	Lymphotoxin alpha	Lta	-4.4*	
Th1	Lymphotoxin beta	Ltb	− 2.0*	
Th17 related	Nicotinamide phosphoribosyltransferase	Nampt	− 2.3*	
Th17	Patelet factor 4 (Cxcl4)	Pf4	− 15.5*	
Th1	Tumor necrosis factor	Tnf	+ 5.3*	
	Tumor necrosis factor receptor superfamily	Tnfrsf11b	− 4.9*	
B stim	Tumor necrosis factor receptor superfamily	Tnfsf13b	− 5.5*	+ 10.3**
Th1 related	Vascular endothelial growth factor A	Vegfa	− 3.0*	

Significant differences between the CD163+/+ and CD163−/− groups are indicated by *p < 0.05, **p < 0.01 (Student’s t-test). ^a^The designation of M1/M2 and Th1/Th2 mediators was mainly based on refs^12,16,24,48–55^. However, it should be noted that many cytokines and chemokines are pleiotropic and possess dual/overlapping functions and the overall cytokine milieu/balance often is of greater importance than the actions of one or two cytokines.

### Synovial inflammatory cell infiltrates in CIA

**Figure 4:**
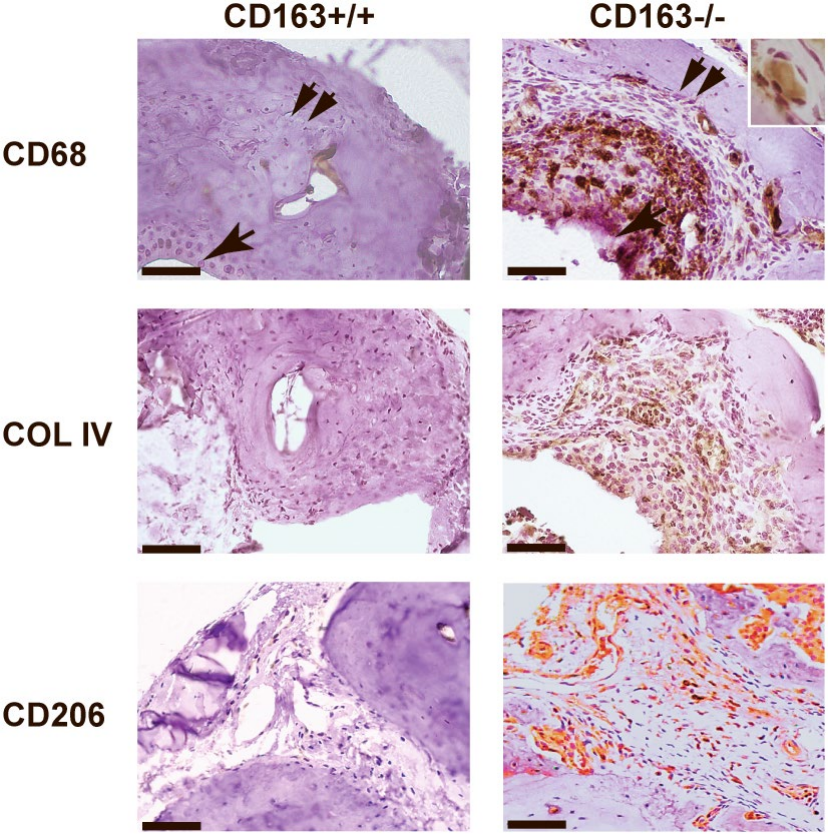
Macrophage infiltration of paw joints in the CIA model. Representative immunohistochemical CD68, Collagen IV and CD206 stainings of formalin fixed and paraffin embedded joint sections in CD163+/+ (n = 10) and CD163−/− mice (n = 11). The single arrow indicates synovial membrane and the double arrow indicates the joint cartilage. Enlarged insert shows the presence of multinucleated CD68 macrophages likely to represent osteoclasts. Magnification × 20. Scale bars equals 50 µm.

**Figure 5:**
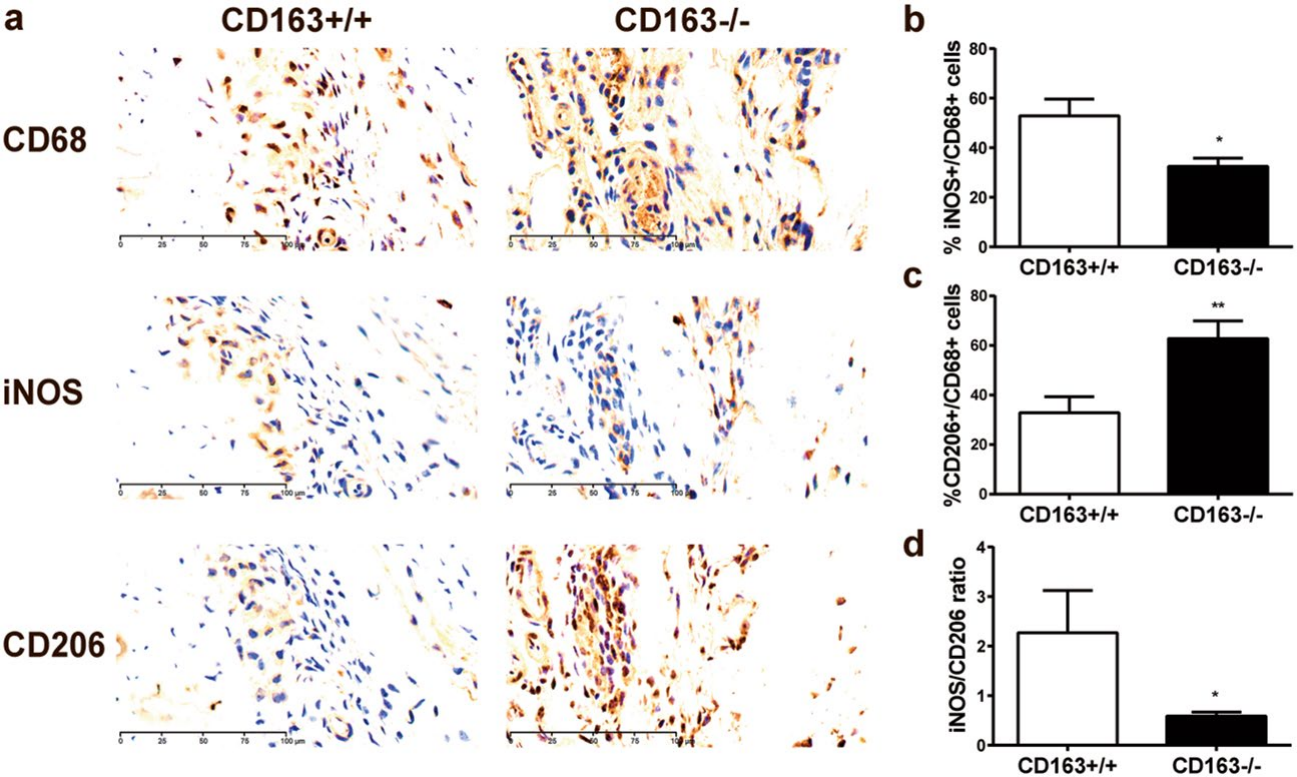
Quantification of M1- and M2-type macrophages in the inflamed joints in the CIA model. (**a**) Representative CD68 (M1 + M2), iNOS (M1) and CD206 (M2) stainings of serial joint sections shown at × 40 magnification. Scale bar equals 100 µm. (**b–d**) Quantitative analysis showing the ratio of iNOS + /CD68 + (**b**), CD206 + /CD68 + (**c**) and iNOS + /CD206 + cells (**d**). Measurements of paw sections from CD163+/+ (n = 6) and CD163−/− mice (n = 6) were made in areas of inflammation at × 20 magnification (3 frames per section). Unpaired t-test performed for significance; *p < 0.05, **p < 0.01 versus wildtype.

**Figure 6:**
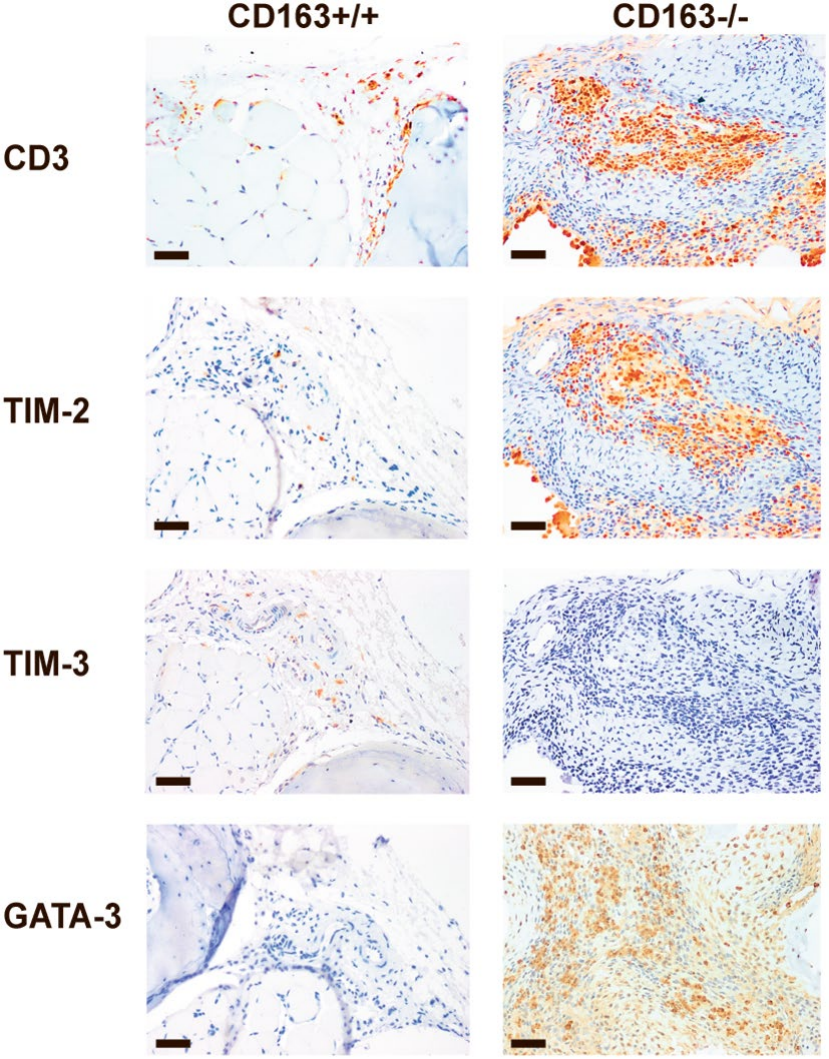
T cell infiltration and T cell subtypes in the CIA model. Representative formalin fixed and paraffin embedded joints sections stained with CD3 antibody recognizing the total pool of T cells and TIM-2 and TIM-3 antibodies specifically binding to the T cell helper cells Th1 and Th2, respectively. Staining of the Th2 specific transcription factor GATA-3 verifies the presence of Th2 cells in CD163-deficient mice. Magnification × 20. Scale bars equals 50 µm. Each staining was repeated with sections from 8 CD163+/+ and 10 CD163−/− mice, respectively.

Immunohistochemical analyses showed marked infiltration of CD68-positive macrophages (Fig. 4) in the synovial membrane of the joints of the CD163−/− mice as well as severe cartilage destruction was evident (Fig. 4). The infiltrating macrophages expressed the mannose scavenger receptor (CD206) (Fig. 4), specifically expressed in M2-type macrophages and dendritic cells. It is involved in collagen internalization and degradation^29^ with an overall anti-inflammatory effect in line with M2-type macrophage expansion in several experimental arthritis models^30^. Quantitative analysis of CD68 (M1 + M2), CD206 (M2) and iNOS (M1) staining of inflamed joints (Fig. 5) further supported predominant M2-type macrophage responses in the CD163−/− mice (Fig. 5c; *p* = 0.006) and predominant M1 responses in the CD163+/+ mice (Fig. 5b; *p* = 0.023). This indicates that the CD163 deficient mice have a strong resolution phase along with and in response to the strong inflammation activity. The presence of multinucleated CD68-positive macrophages (Fig. 4), as well as the detection of tartrate-resistant acid phosphatase (TRAP) activity in multinucleated osteoclast-like cells (Supplementary Fig.S2), suggests pronounced osteoclast bone reabsorption and remodeling in the CD163−/− mice. Further, CD3 staining of the joints showed high influx of T cells in the CD163−/− mice compared to the wild-type mice (Fig. 6). The majority of T cells expressed the T cell immunoglobulin domain and mucin domain 2 (TIM-2) in the CD163−/− mice in line with up-regulation of Th2 cell responses. The presence of the GATA-binding protein3 (GATA-3) (Fig. 6), which is necessary for Th2 differentiation also supports prevalent Th2 responses in the CD163−/− mice.

### Systemic responses in CIA

In accordance with the findings in the joints, the serum level of the Th1-related cytokine IFN-γ was lower in the CD163−/− mice (Table 2; *p* = 0.047), whereas the Th2-related macrophage inflammatory protein (MIP3a)/ CCL20) was highly up-regulated compared to the wild-type mice (Table 2; *p* < 0.0001). Serum TNF-α was lower in the CD163−/− mice (Table 2; *p* = 0.042), albeit RNA expression of TNF-α was up-regulated locally in the inflamed joints of the CD163−/− mice (Table 1). Serum levels of the acute-phase protein Hp, (and CD163 ligand when complexed to hemoglobin), were higher in the CD163−/− mice compared to wild-type mice (Table 2; *p* = 0.029), whereas hemoglobin levels were lower in the CD163−/− mice (Table 2; *p* = 0.016). Further, Hp levels were elevated in arthritic mice compared to naïve mice, and the serum Hp correlated with disease severity at termination of the study (*p* = 0.01, R = 0.32). The spleen, which also is a well-known indicator of systemic inflammation, was significantly larger in the CD163−/− mice compared to wild-type mice (Table 2; *p* = 0.009).

**Table 2:** Local and systemic inflammatory characteristics in CD163+/+ and CD163−/− mice.

	Naïve CD163+/+ (n = 9)	Naïve CD163−/− (n = 10)	CIA CD163+/+ (n = 8–18)	CIA CD163−/− (n = 9–21)
**Joint levels**				
Hemoglobin (mg/ml)			0.18 ± 0.06	0.24 ± 0.03
Haptoglobin (µg/ml)			0.29 ± 0.03	0.72 ± 0.17*
**Synovial fluid levels**				
Hemoglobin (mg/ml)			0.22 ± 0.07	0.16 ± 0.04
Haptoglobin (µg/ml)			15.50 ± 5.20	91.50 ± 31.5*
TNF-α (pg/ml			161.2 ± 23.9	87.8 ± 6.6*
**Systemic serum levels**				
Hemoglobin (mg/ml)	0.60 ± 0.04	0.56 ± 0.02*	1.15 ± 0.23	0.49 ± 0.25*
Haptoglobin (µg/ml)	11.76 ± 32.2	24.30 ± 9.7	94.3 ± 24.1	194.4 ± 38.7*
CCL20/MIP3α (pg/ml)			39.1 ± 4.5	85.6 ± 9.7****
INF-γ (pg/ml)			2.1 ± 0.2	1.7 ± 0.2*
TNF-α (pg/ml)			124.0 ± 27.2	68.9 ± 6.9*
**Spleen weight** (g)			0.076 ± 0.002	0.095 ± 0.005**

Values are means ± SEM. Unpaired t-tests for significance. Significant differences between male CD163+/+ and CD163−/− groups are indicated by *p < 0.05, **p < 0.01 and ****p < 0.0001.

### M1 and M2 gene signature in CD163−/− mice

To analyze if the increased severity of CIA in CD163−/− mice was due to a difference in the phenotype of the macrophages and sensitivity to external stimulation, we analyzed the expression of M1 and M2 gene signatures in in vitro*-*stimulated bone marrow derived macrophages (BMDM) from wild type and CD163-deficient mice. BMDM were differentiated in vitro for 7 days and following stimulated with either IL4, IL10, GM-CSF or IFNγ for 8 h (Supplementary Fig.S3). Gene expression analysis showed the expected upregulation of primarily M1-associated genes in IFNγ and GM-CSF stimulated cells and upregulation of M2-associated genes in BMDMs stimulated with IL4 and IL10. No difference was observed between wild type and CD163−/− mice. We then explored if the increase in arthritis severity was caused by a CD163-dependent defect in the T cell compartment. CD4^+^ naïve T cells from wild type and CD163−/− mice were isolated from spleen and subjected to in vitro- differentiation into either Th1, Th2 or Th17 cells. Gene expression analysis (Supplementary Fig.S3) showed a specific upregulation of Th1-, Th2- and Th17-specific gene signatures in Th1-, Th2- and Th17-differentiated T cells, respectively. The signatures were similar in CD163−/− mice and CD163+/+ mice indicating that CD163-defiency does not alter the intrinsic ability of CD4^+^ naïve T cells to differentiate into Th1, Th2 or Th17 cells.

### Development of collagen antibody-induced arthritis (CAIA) in CD163+/+ versus CD163−/− mice

**Figure 7:**
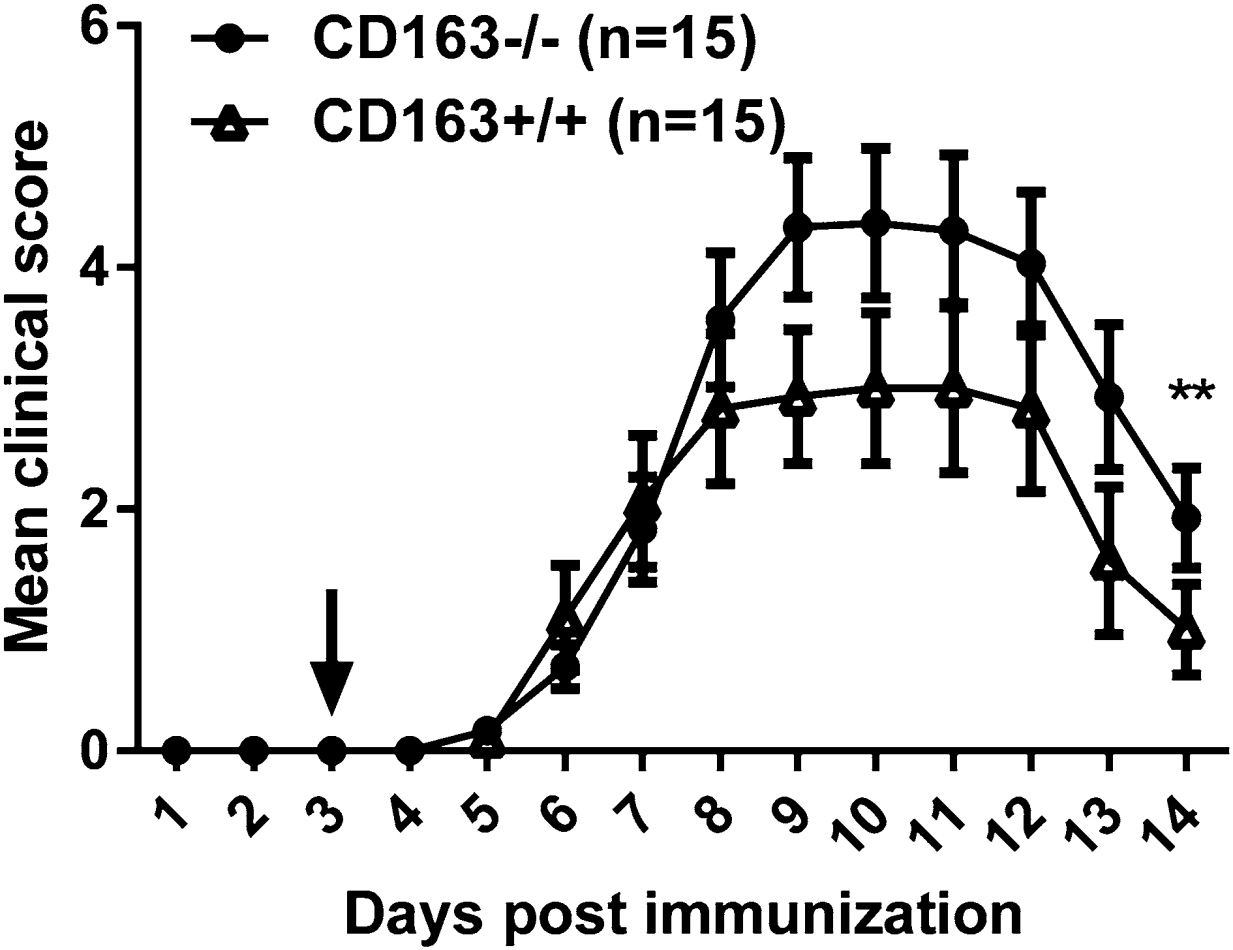
Development of CAIA in wild-type and CD163−/− mice. Clinical scores of wildtype and CD163−/− following i.v. injection of a cocktail of anti-collagen type II antibodies. Arrow indicates LPS boost injection. N = 15 of each group. Values are means ± SEM. Paired t-test for significance;** *p* < 0.01 versus wildtype. The figure is representative of three independent experiments.

The CAIA model, which is a more simple and largely T cell-independent model of rheumatoid arthritis, was used to further address the effect of CD163 deficiency during inflammatory disease^31,32^. Arthritis was induced by systemic administration of a cocktail of monoclonal antibodies directed to conserved epitopes found on collagen type II, followed by an injection of LPS. The clinical disease severity was only slightly more pronounced in CD163−/− compared to the wild-type mice (Fig. 7; *p* = 0.007).

## Discussion

In the present study, we observed a much more severe development of CIA in C57BL/6 mice deficient in CD163 expression compared to wild-type C57BL/6 mice that have a genetic background characterized by a general low susceptibility to collagen immunization in terms of arthritis development. In contrast, a similar difference was not seen in the CAIA model, wherein arthritis was induced with collagen antibodies directly. In addition to the strong anti-inflammatory effect of CD163 on disease activity in the CIA mice, we observed a major difference in the apparent Th1/Th2 and M1/M2 balance, when comparing the collagen autoantibody IgG subclasses, T cell and macrophage phenotypes as well as cytokine profiles of the collagen-immunized CD163−/− mice versus CD163+/+ mice. Surprisingly, the enhanced arthritis disease in the CD163−/− mice was not accompanied by an apparent increased Th17 response.

Previous studies have shown that development of CIA is highly dependent upon a Th1 and Th17 responses in C57BL/6 mice and it is generally assumed that the induction phase of CIA is characterized by a Th1-like cytokine response, whereas Th2 cytokines are suggested to have a more prominent role during the remission phase of the disease^25,33,34^. T cell responses are known to be Th1-related 6–8 weeks after onset of arthritis in wildtype C57BL/6 mice^35^. On the other hand, mouse strains like BALB/c that have a predominant Th2 response are much more prone to develop severe arthritis compared to C57BL/6 which is generally assumed to be predisposed to Th1^36^. Thus, Th2 cytokines may play a pro-inflammatory role. In this view, it is intriguing that we see a predominant Th2 response in the CD163−/− mice after 52 days when the mice still have a strong and fulminant disease. Thus, the increased inflammation and joint destruction in the CD163−/− mice is likely to reflect a chronic and progressive inflammation characterized by pro-inflammatory Th2 responses rather than reflecting a remission phase. Along this line, the upregulated cytokines IL-7 and IL-33 are potent pro-inflammatory Th2 mediators involved in osteoclasts differentiation, activation of synovial fibroblast and mast cells within the arthritic joints^37–40^. This is further supported by the increased expression of GATA-3, which is critical for Th2 differentiation and proliferation^41^, locally in the synovium in the CD163−/− mice. Th2 cells are involved in eosinophil recruitment and secretion of interleukin IL-3, IL-4, IL-5, and IL-13^42^. However, the expression of these genes was not significantly up-regulated, and instead IL-3 and IL-5, known to be positively correlated with blood eosinophil number, were noticeable down-regulated in the CD163−/− mice. RNA up-regulation of CCL22 supports Th2/Treg responses, whereas RNA expression of CCL24 and CCL11 implicated in Th2/Treg recruitment and eosinophil function were down-regulated.

The molecular mechanism(s) for the enhanced inflammatory response and the increased Th2 activity by CD163 inactivation is unknown and needs further investigation, but it is likely to relate to a function of the M2-type macrophages, which is the major cell type for CD163 expression that exclusively is expressed in the myelo-monocytic linage. Here we show an increased number of macrophages expressing CD206 as well as M2 responses in the inflamed joints of CD163−/− mice compared to the CD163+/+ mice. Importantly, CD163 deficiency did not affect CD206 expression in bone marrow derived macrophages indicating that the altered macrophage activity/phenotype in CD163−/− mice is not a result of an intrinsic defect in the macrophages. Although M2 macrophages are termed ‘anti-inflammatory’ they also contribute largely to maintaining inflammation, e.g. by production of pro-inflammatory cytokines. This inflammation-stabilizing role of the M2 macrophages is for instance indicated by the strong reducing effect on the TNF-α, IL-1β and IL-6 plasma concentration when targeting anti-inflammatory glucocorticoids to CD163 positive M2 cells in endotoxin-treated rats and pigs^43,44^. It is possible that the anti-inflammatory effect of CD163 in arthritis as well as the changed Th1/Th2 balance relates to the function of CD163 as an endocytic receptor in the macrophages. The receptor is an established receptor for uptake of Hb released into plasma (free or in complex with Hp)^1^ and this function is regarded to serve an anti-inflammatory role because it removes pro-inflammatory heme/Hb and transforms it into the anti-inflammatory metabolites bilirubin and CO^45^. CD163 is also reported as a receptor for uptake of other pro-inflammatory substances such HMGB1 (also bound to Hp)^7^ and TWEAK^6^. However, the endocytosis of any of these ligands does not readily explain the strong effect on the change in Th1/Th2 balance and the increase in arthritis disease severity in the CD163-deficient mice in the present study. Neither was increased hemoglobin metabolism indicated by signs of intraarticular bleeding. We therefore find it is tempting to speculate that CD163 serves an additional anti-inflammatory function by removal of yet unknown ligand (s) somehow regulating naïve T cells and the Th1/Th2 response, because CD163 deficiency per se does not affect in vitro Th1, TH2 og Th17 differentiation of CD4 cell. Most endocytic receptors are multi-ligand receptors and yet unknown pro-inflammatory ligands to CD163 might therefore well remain to be discovered. In conclusion, the present data demonstrate a strong anti-inflammatory role of CD163 that affects the T-cell response.

Furthermore, the present data provide a new model for studying experimental arthritis in gene knock-out models by breeding into CD163 knock-out mice or by inactivation of CD163 expression/function by other means (e.g. CRISP-Cas). Since most gene knock-out mouse models are on a C57/BL6 background and therefore rather resistant to CIA, it will be straightforward to investigate other genes involved in chronic CIA inflammation using the CD163-deficient CIA model. This may further define molecular mechanisms and signaling pathways that drive T cell as well as macrophage polarization in experimental and human autoimmune arthritis. It would also be highly relevant to further investigate the role of the MHC class II subtypes (such as I-Aq, I-Ar and H-2q not present C57BL/6) that are regarded as important for susceptibility to collagen-induced arthritis in mice.

## Methods

### Animals

CD163 knockout mice (Cd163-/-;CD163tm1(KOMP)Vlcg) were generated on a C57/BL6 background using targeting constructs available from the University of California at Davis International Mouse Consortium (KOMP) as previously described ^3^. Animals for establishing a CD163−/− breed is available from this institution. Wild-type mice on a C57/BL6 genetic background were used for all experiments including litter-mate controls. The animals were maintained under controlled temperature (20 ± 2 °C) and light (lights on 08:00–20:00 h) with acces to water and food ad libitum. One week before induction of arthritis, the mice were allocated at random to cages according to standard requirements. The experimental protocols were approved by The Danish Experimental Animal Inspectorate (permission no. 2014-15-0201-00240) and experiments were performed in accordance with relevant guidelines and regulations of the institutions (Aarhus University and the University of Southern Denmark).

### Induction of collagen-induced arthritis (CIA)

For induction of CIA, 10- to 12-wk old CD163−/− (n = 15), CD163+/− (n = 7) and CD163+/+ (n = 12) male mice (three independent experiments) were immunized subcutaneously at the base of the tail with 200 μg of chicken collagen type II (CII) (Chondrex Inc., Redwood, WA, USA) emulsified in Freund's Complete Adjuvant (CFA) containing 150 µg heat inactivated M. tuberculosis, and boosted with 100 μg of CII in Freund's Incomplete Adjuvant (IFA) at day 21. CII was dissolved overnight (ON) at 4 °C in 10 mM acetic acid. From day 15, the mice were scored three times per week for clinical signs of arthritis and each of the 4 limbs were assessed by a clinical score of 0–4 (modified from^14^, thus giving a total score between 0 and 16; 0 = normal, 0.5 = light redness of ankle or digits , 1 = mild, but definite redness and swelling of the ankle or wrist, or apparent redness and swelling limited to individual digits, regardless of the number of affected digits, 2 = moderate redness and swelling of ankle and wrist, 3 = severe redness and swelling of the entire paw including digits and 4 = maximally inflamed limb with involvement of multiple joints Fifty-two days after the first immunization with CII, the mice were sacrificed following retro-orbital bleeding and tissue samples were collected and prepared as described below.

### Collagen antibody-induced arthritis (CAIA)

CAIA was induced by intravenous injection of 4 mg of a 5-clone antibody cocktail (recognizing conserved epitopes) of anti-collagen antibodies (Chondrex Inc.) in CD163−/− (n = 15) and wildtype mice (n = 15) male mice (13–15 weeks, three independent experiments)^19^. Disease progression was boosted by injection of LPS (40 µg/mouse) 3 days post the antibody injection. Disease development and severity was determined daily as described above.

### Serum and tissue preparation

At termination of the studies, the mice were anesthetized with isoflurane and blood samples were collected by retro-orbital bleeding before killing by cervical dislocation. Serum was prepared by centrifugation of coagulated blood samples at 4,000 rpm for 20 min and frozen at -20 °C. Tissue samples for RNA and protein preparation was snap-frozen in liquid nitrogen and stored at -80 °C. Whole joints including synovium, adjacent tissues and bones were homogenized in lysis buffer using a Polytron homogenizer and the protein extracts were stored at -80 °C until further analysis. Paws for histology and spleens were fixed in 10% neutral buffered formalin. One week later, the paws were transferred to 15% EDTA pH 7.4 for decalcification before histopathological examination.

### Histological analysis

Paraffin-embedded Sects. (5 µm) of the paws were prepared and then stained using hematoxylin–eosin (HE) to evaluate pannus formation, synovial cell infiltrate and cartilage/bone erosion. For immunohistochemical studies of the joints, the sections were incubated with 5% goat serum for 30 min at RT. After rinsing and heat-induced epitope retrieval, the sections were incubated ON at 4 °C with rat anti-mouse TIM2 (Biorad, Oxford, UK), rat anti-mouse TIM3 (Biorad), rat anti-mouse CD3 (Biorad), rabbit anti-mouse CD4 (Abcam, Cambridge, UK), rabbit anti-mouse CD68 (Abcam), rabbit anti-mouse IL17A (NJS Bioreagents, San Diego, CA), (rabbit anti-mouse CD206 (Abcam), rabbit anti-mouse CD163 (Abcam), iNOS (Abcam), rabbit anti-mouse GATA3 (Abcam) and rabbit anti-mouse collagen IV (Abcam) antibodies. Blocking of endogen peroxidase was performed in 1.5% H_2_0_2_ for 10 min before incubation with the secondary antibodies donkey anti-rat IgG HRP (Abcam) or goat anti-rabbit IgG HRP (Abcam) for 2 h at RT. Quantitative measurements of CD68, CD206, iNOS and IL17A staining were determined using ImageJ software (NIH). Staining of tissue sections for osteoclast activity was performed by a leukocyte acid phosphatase kit, a cell-staining kit for the detection of tartrate resistant acid phosphatase (TRAP) from Sigma-Aldrich (Copenhagen, Denmark). Images were taken using an Olympus CKX41 microscope with a DP27 camera.

### Measurement of paraclinical parameters

Serum levels of mouse type II collagen antibodies (Chondrex Inc.), TNF-α (eBioscience, San Diego, CA, USA), IFN-γ (Abcam) and CCL20 (Abcam), Hp (Abcam) and Hb (Abcam) were determined in CIA mice using commercial enzyme-linked immunosorbent assays (ELISAs) according to the manufactures recommendations.

### Cell culture

Bone marrow derived macrophages (BMDM) and CD4 + naïve T cells were obtained from 10- to 12-wk old CD163−/− (n = 5) and CD163+/+ (n = 5). For BMDMs, femurs and tibiae were flushed and cells collected by centrifugation at 400 g for 5 min at 4 °C. Cells were resuspended in RPMI1640 supplemented with L-glutamine (2 mM), penicillin (100 U/ml)/streptomycin (100 mg/ml) (Gibco), 10% heat-inactivated FCS and 10% L929 conditioned medium, counted and cultured at a density of 2 × 10^6^ cells/ml in non-tissue culture treated plastic dishes (BD Pharmingen) at 37 °C and 5% CO2. After 7 days, adherent cells were collected and resuspended in complete RPMI1640 containing 10% L929 conditioned media and stimulated with 10 ng/µl IFNg, IL10, GM-CSF or IL4 (all Peprotech Nordic, Stockholm, Sweden) for 8 h. CD4^+^ naïve T cells were isolated by negative selection from single cell suspensions of splenocytes using the EasySep Mouse CD4 + T cell isolation kit (STEMCELL Technologies, Cambridge, UK). In vitro differentiation of Th1, Th2 and Th17 T cells were following done in 24 well plates precoated with anti-CD3e antibodies (clone 2C11, Bioxcell, US) exactly as described in^46^. For subsequent in vitro differentiation, CD4^+^ naïve t-cells were resuspended in RPMI1640 supplemented with 10% FBS, NEAA, antibiotics, and 55 μM β-mercaptoethanol and incubated for 96 h at 37 °C, 5% CO_2_ with Th1, Th2 and T17 supplements as follows; Th1: 0.5 μg/mL anti-CD28 (clone PV1.17, Bioxcell) and 1 μg/mL anti-IL-4 antibodies (Clone 11B11, Bioxcell), 5 ng/mL IL-2, and 10 ng/mL IL-12 (Peprotech); Th2, 0.5 μg/mL anti-CD28 and 1 μg/mL anti-IFN-γ antibody (Clone R4-6A2, Bioxcell) , 5 ng/mL IL-2, and 10 ng/ mL IL-4 (peprotech); Th17, 0.5 μg/mL anti-CD28, 1 μg/mL anti-IFN-γ, 1 μg/mL anti-IL-2 (Clone JES6-1A12, bioxcell), and 1 μg/mL anti-IL-4 antibodies, 10 ng/mL mouse IL-6, and 1 ng/mL TGF-β1 (Cell signaling, MA, US).

### Gene expression profiling

Total RNA was purified using the RNeasy Mini Kit (Qiagen, København Ø, Denmark). RT^2^ Profiler™ PCR Arrays with 92 arthritis biomarkers were designed for Fluidigm® BioMark™ analysis (see Supplementary Excel file for target and primer details). Reverse-transcription of RNA and preamplification of genes of interest was done using the Fluidigm Reverse Transcription Master Mix and the Fluidigm PreAmp Master Mix (Fluidigm Corporation, South San Francisco, CA, USA) in accordance with the manufactures instructions. In short, for initial cDNA synthesis 10 ng of RNA was mixed with the RT mastermix (5x) in a 1:1 ratio and diluted 5 times in RNAse-free H2O followed by incubation at the following conditions: 25 °C for 5 min, 42 °C for 30 min and finally 85 °C for 5 min. To increase sensitivity, genes were pre-amplified by pooling 96 deltagene assays (Fluidigm,) allowing a final concentration of 500 nM per assay. In the final pre-amplification reaction mixture cDNA was diluted 1:4 with Fluidigm PreAmp Master Mix (5X), pooled deltagene assays (10X) and H2O. Reaction mixture was incubated at the following PCR conditions: 95 °C for 2 min followed by 14 cycles of 95 °C for 15 s and 60 °C for 4 min. To remove unincorporated primers pre-amplified cDNA was incubated with 1,25U/µl Exonuclease I at 37 °C for 30 min followed by 80 °C for 15 min. After Exonuclease I treatment pre-amplified cDNA was diluted 1:5 with TE buffer (10 mM Tris–HCl, 1 mM disodium EDTA, pH 8.0) and stored at -20 °C. Gene expression analysis was carried out using the 96.96 dynamic arrays and Biomark HD system from Fluidigm (Fluidigm Europe B.V., Amsterdam, Netherlands). Specifically, a 6 μl sample mixture was prepared for each sample containing 1 × SsoFast EvaGreen Supermix with low ROX (BioRad), 1× DNA Binding Dye Sample Loading Reagent and each of diluted pre-amplified samples. Six μl of Assay mix was prepared with 1× Assay Loading Reagent, 100 µM of each of the different Deltagene Assays and TE buffer. The 96.96 dynamic arrays were primed and loaded in an IFC controller with in between additions of samples and assay mixes in the appropriate inlets. After loading, the chip was placed in the BioMark HD Instrument for an initial thermal mix that included incubation at 70 °C for 40 min followed by 60 °C for 0.5 min. Subsequent qPCR was performed by 1 cycle of hot start at 95 °C for 1 min, followed by 30 cycles at 96° C for 5 s and 60 °C for 20 s with fluorescent recording after each cycle. For analysis of PCR products, the qPCR run was followed by a melting curve analysis where the fluorescent signal from dsDNA was measured at 60 °C-95°C with 1 °C increment. The data was analyzed with Real-Time PCR Analysis Software (Fluidigm Europe B.V.). The RT2 Profiler PCR Array Data Analysis Webportal (www.sabioscience.com) was used to analyze CT values and to calculate changes in gene expression. The relative expression levels of each target gene was normalized to actin- beta, beta-2 microglobulin, glyceraldehyde-3-phosphate dehydrogenase, beta-glucuronidase and heat-shock protein 90 alpha and calculated by the 2-delta delta Ct method^47^.

### Statistical analysis

All statistical analysis was performed in Graphpad Prism 6. All data variables were tested by the Shapiro–Wilk normality test to determine appropriate statistical analysis. Normally distributed variables are presented as mean ± SEM. For comparison of the two groups, parametric data were analysed using two-tailed, paired or unpaired Student’s t-test. Nonparametric data were tested using Kruskal–Wallis test, followed by the Wilcoxon Mann–Whitney Rank sum test. P-values < 0.05 were considered significant.

## Supplementary information

Supplementary Information.

## Data Availability

The data sets generated and/or analysed during the current study are available from the corresponding author on reasonable request.
