# Supplementary material for: Mouse CD163 deficiency strongly enhances experimental collagen-induced arthritis
Source: Sci Rep. 2020 Jul 24;10:12447. doi: 10.1038/s41598-020-69018-7 (PMC7382459; doi:10.1038/s41598-020-69018-7)
Supplement: Supplementary file 1 — Supplementary Information. [file 41598_2020_69018_MOESM1_ESM.docx]

**Mouse CD163 deficiency strongly enhances experimental collagen-induced arthritis**

Pia Svendsen^1,2^, Anders Etzerodt^3^, Bent Deleuran^3,4^ and Søren K. Moestrup^1,3,5*^.

^1^ Institute of Molecular Medicine, University of Southern Denmark, Denmark

^2^ Department of Clinical Medicine, Aarhus University Hospital, Aarhus Denmark

^3^ Department of Biomedicine, Aarhus University, Aarhus Denmark

^4^ Department of Rheumatology, Aarhus University Hospital, Aarhus Denmark

^5^ Department of Clinical Biochemistry and Pharmacology, Odense University Hospital, Denmark

**Supplementary Data**

**
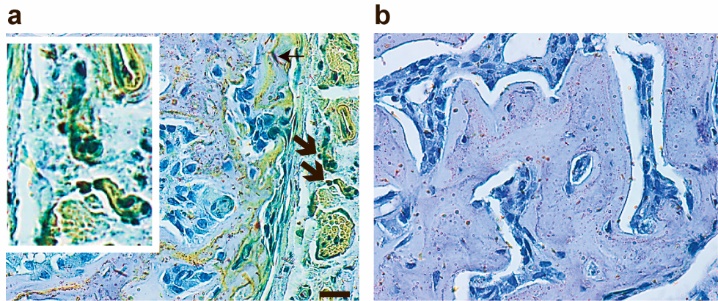
**

**Supplementary Figure S1. (a)** Tartrate-resistant acid phosphatase (TRAP) staining on paraffin section of the joint of CD163-/- mice, showing multinucleated giant cells (arrows; detail in inset) staining positive for TRAP. Yellow - TRAP+ multinucleated cells (three or more nuclei) were considered to be osteoclasts. **(b)** Control staining without addition of the Tartrate solution to the acid phosphatase test solution provided by Sigma-Aldrich. Magnification x40. Scale bar equals 20 µm.


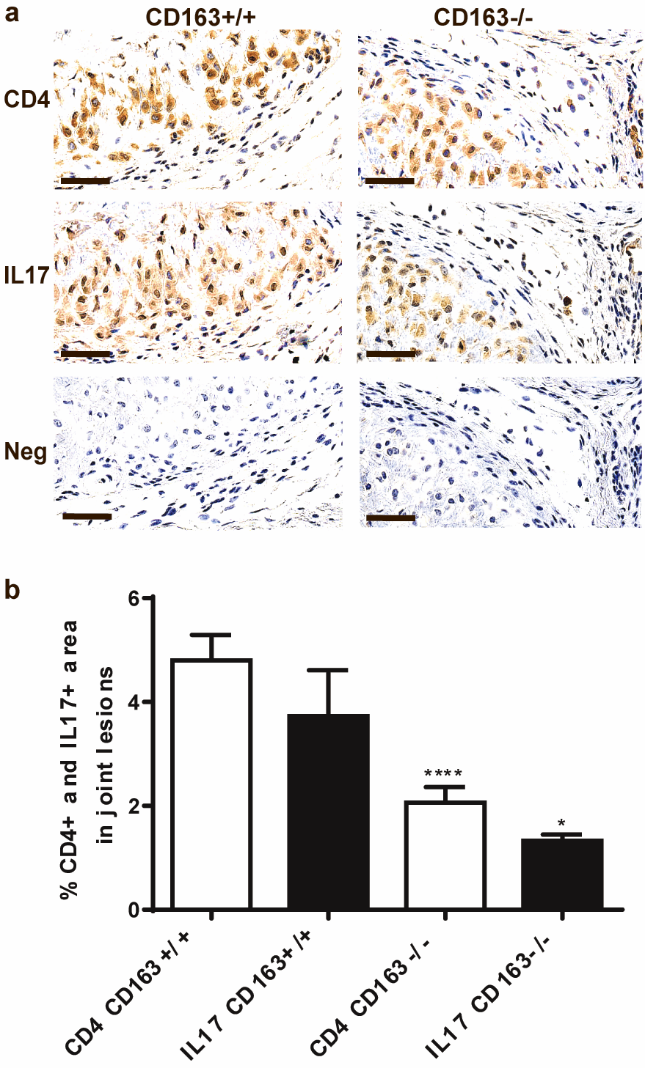


**Supplementary Figure S2**. Quantification of CD4 and IL17A T cells in the inflamed joints in the CIA model. (**a**) Representative CD4 and IL17A stainings of serial joint sections shown at x20 magnification. Scale bar equals 50 µm. (**b**) Staining area measurements of paw sections from CD163+/+ (n=6) and CD163-/- mice (n=6) were made in areas of inflammation at x20 magnification (2 frames per section). Unpaired t-test performed for significance; *p< 0.05, ****p< 0.0001 versus wildtype.

**
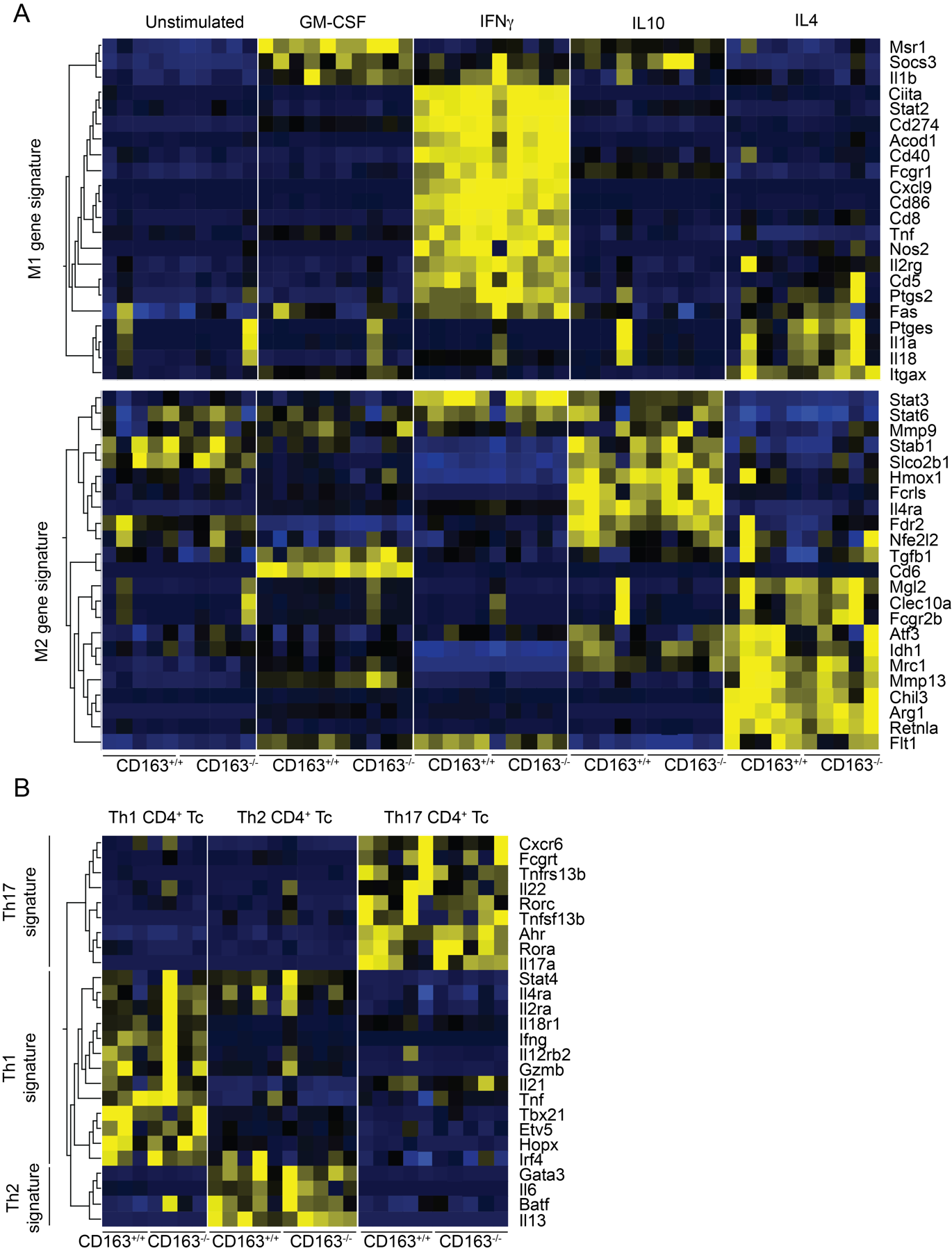
**

**Supplementary Figure S3.** Gene expression profile of BMDM and T cells from CD163 +/+ or CD163 -/- mice after in vitro stimulation. **(a)** Expression of a M1 or M2 gene signature in in vitro stimulated BMDM isolated from CD163 +/+ (n=5) or CD163 -/- mice (n=5). BMDMs where either unstimulated or stimulatied with GM-CSF, IFNg, IL10 or IL4 for 8 hrs prior to analysis. **(b)** Expression of a Th1, Th2 or Th17 gene signature in Th1, Th2 or Th17 cells after in vitro differention from CD4+ naïve T cells isolated from CD163 +/+ (n=5) or CD163 -/- mice (n=5).

**
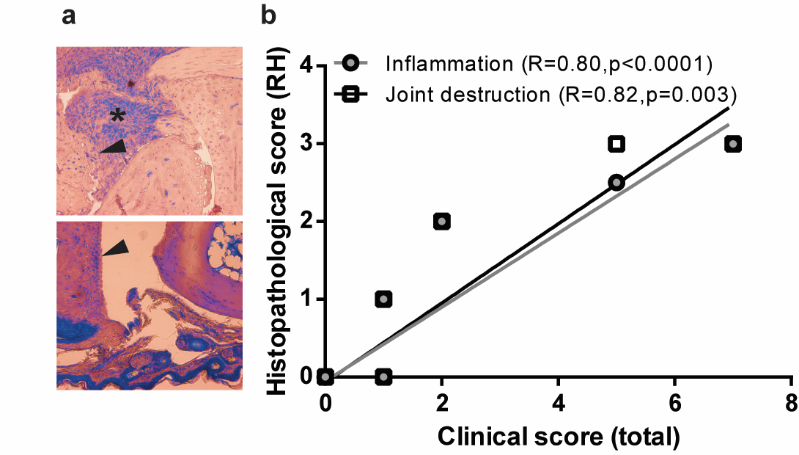
**

**Supplementary Figure S4.** Development of CAIA in wildtype and CD163-/- mice. **(a)** Histopathological sections of the joints of arthritic mice with a high score (top) and low score (bottom), respectively. Asterisk indicates pannus formation and single arrow indicates the synovial membrane. **(b)** Correlation between the clinical scores and the histopathological inflammation and joint destruction scores of the right hind paws (RH), respectively of mice with CAIA.
